# Interfollicular epidermal stem-like cells for the recreation of the hair follicle epithelial compartment

**DOI:** 10.1186/s13287-020-02104-9

**Published:** 2021-01-15

**Authors:** Carla M. Abreu, Rogério P. Pirraco, Rui L. Reis, Mariana T. Cerqueira, Alexandra P. Marques

**Affiliations:** 1 3B’s Research Group – Biomaterials, Biodegradables and Biomimetics, Headquarters of the European Institute of Excellence on Tissue Engineering and Regenerative Medicine, University of Minho, Guimarães, Portugal; 2 ICVS/3B’s – PT Government Associate Laboratory, Braga/Guimarães, Portugal

**Keywords:** Epidermal keratinocytes with stem-like features, Dermal papilla cells, Epithelial-mesenchymal interactions, Hair follicle, Sebaceous gland

## Abstract

**Background:**

Hair follicle (HF) development and growth are dependent on epithelial-mesenchymal interactions (EMIs). Dermal papilla (DP) cells are recognized as the key inductive mesenchymal player, but the ideal source of receptive keratinocytes for human HF regeneration is yet to be defined. We herein investigated whether human interfollicular epidermal keratinocytes with stem-like features (EpSlKCs), characterized by a α6^bri^/CD71^dim^ expression, can replace human hair follicular keratinocytes (HHFKCs) for the recreation of the HF epithelium and respective EMIs.

**Methods:**

The α6^bri^/CD71^dim^ cellular fraction was selected from the whole interfollicular keratinocyte population through fluorescence-activated cell sorting and directly compared with follicular keratinocytes in terms of their proliferative capacity and phenotype. The crosstalk with DP cells was studied in an indirect co-culture system, and EpSlKC hair forming capacity tested in a hair reconstitution assay when combined with DP cells.

**Results:**

EpSlKCs exhibited a phenotypic profile similar to follicular keratinocytes and were capable of increasing DP cell proliferation and, for short co-culture times, the number of alkaline phosphatase-active cells, suggesting an improvement of their inductivity. Moreover, the recreation of immature HFs and sebaceous glands was observed after EpSlKC and DP cell co-grafting in nude mice.

**Conclusions:**

Our results suggest that EpSlKCs are akin to follicular keratinocytes and can crosstalk with DP cells, contributing to HF morphogenesis in vivo, thus representing an attractive epithelial cell source for hair regeneration strategies.

## Background

The hair follicle (HF) singular capability to continually self-renew and undergo repeated cycles relies on the reciprocal interactions that occur between its mesenchymal and epithelial compartments [1, 2]. Therefore, successful strategies aiming to promote HF regeneration rely on the combination of relevant dissociated cell populations to rescue epithelial-mesenchymal interactions (EMIs) [3, 4]. The HF mesenchyme is usually recreated with specialized dermal papilla (DP) cells, whereas the epithelial fraction is commonly reconstructed using keratinocytes (KCs) isolated from different follicular sources, including the bulge [5], the outer root sheath (ORS) [6–10], or the hair bulb [8, 11]. Yet, the ideal epithelial source is not identified. Although representing a stem cell niche, the bulge can only be accessed from intact HF [12], which is hindered by fairly indistinguishable anatomical boundaries [13]. Hence, KCs are frequently obtained from the hair bulb or ORS of plucked HFs, an easier access source. However, their isolation relies on the rough HF transection, followed by enzymatic digestion or cell outgrowth [12, 14, 15]. This represents a low yield process [16] where population purity is reduced due to the contamination with other cell fractions. Moreover, the isolated cells are at distinct differentiation stages, most of them being transit-amplifying (TA) cells, with shorter culture lifespan in comparison to stem cells [12].

Human interfollicular epidermal cells have also been used for HF regeneration, but successful strategies rely on their combination with murine DP cells [17] that better retain inductivity than human cells. Neonatal epithelial cell sources are potentially the most useful due to the immature state of the cells; however, accessibility and ethical constraints still feed the gap between demand and supply. Knowing that epidermal stem cells express high levels of the α6-integrin (also known as CD49f) and can be distinguished from other basal cells based on their lower expression of the transferrin receptor (CD71) [18, 19], we previously demonstrated that the α6^bri^CD71^dim^ subpopulation of interfollicular epidermal stem-like cells can be isolated from adult skin [19]. Considering their undifferentiated state and high clonogenic capacity [19], we hypothesized that these epidermal KCs with stem-like features (EpSlKCs) can replace follicular keratinocytes in the recreation of the HF epithelium and respective EMIs with DP cells, promoting HF formation. EpSlKCs were isolated by fluorescence-activated cell sorting (FACS) based on their α6^bri/^CD71^dim^ profile, and their proliferative capacity and phenotype were directly compared with the commonly used human hair follicular keratinocytes (HHFKCs). Next, EpSlKC capacity to affect and communicate with DP cells was tested in indirect co-cultures focusing on cell proliferation, phenotype, and release of the HF-related growth factors PDGF-A, VEGF, and BMP2. Finally, EpSlKC hair trichogenic capacity was assessed in a hair inductive assay in combination with human DP cells.

## Methods

Human skin was obtained from abdominoplasties, performed at Hospital da Prelada (Porto, Portugal), while human occipital scalp samples from hair transplantation surgeries at Sanare Unicapilar (Porto, Portugal). Samples were obtained under a collaboration protocol approved by the ethical committees of the involved institutions and after written informed consent from patients. Animal experimentation was approved by the local Animal Welfare Body.

### Cell isolation and culture

KCs were isolated as previously described [19] and cultured in keratinocyte serum-free medium (KSFM, Gibco) supplemented with 1% penicillin-streptomycin solution (PenStrep, Gibco) and with the ROCK pathway inhibitor Y-27632 (10 μM, STEMCELL Technologies). HHFKCs were purchased from ScienCell (CA, USA) and cultured in the supplied keratinocyte medium. DPs were microdissected [20] from occipital scalp samples. The explants were cultured in Dulbecco’s modified Eagle’s medium (DMEM, Sigma-Aldrich) supplemented with 20% FBS and 1% antibiotic-antimycotic solution (Gibco). Cells growing out of the explant were further expanded in collagen (Sigma-Aldrich)-coated flasks and cultured in DMEM with 10% FBS and 1% antibiotic-antimycotic solution. Cell culture was conducted under standard conditions (37 °C, 5% CO_2_) in a humidified atmosphere and with medium change every 2–3 days.

### Feeder layer preparation

J2-3T3 mouse fibroblasts were cultured in DMEM with 10% bovine serum (Life Technologies). Cells were then inactivated with mitomycin C (4 μg/ml, Sigma-Aldrich) for 2 h 30 min at 37 °C [21]. Feeder layers were prepared by seeding the inactivated 3T3 fibroblasts at a density of 2.4 × 10^4^ cells/cm^2^.

### Selection of the α6^bri^/CD71^dim^ cellular subpopulation

A suspension of primary KCs with 5.0 × 10^6^cells/ml was incubated with CD71-PE (1:200, BioLegend) and CD49f-APC (1:85, eBioscience). The selection of α6^bri^/CD71^dim^ cells was performed using a FACSAriaIII Cell Sorter and FACSDiva software (BD Biosciences). Sorted α6^bri^/CD71^dim^ cells were plated at 1.8 × 10^3^ cells/cm^2^ onto the feeder layer and cultured in FAD medium [1-part Ham’s F-12 (Sigma-Aldrich) and 3-parts DMEM supplemented with 10% non-inactivated FBS, 1.8 × 10^4^ M adenine, 0.5 μg/ml hydrocortisone, 5 μg/ml insulin, 10^–10^ M cholera toxin (Sigma-Aldrich), 10 ng/ml epidermal growth factor (Peprotech), 1.8 mM CaCl_2_ (Merck), and 1% PenStrep].

### Flow cytometry analysis

For surface marker analysis, cell suspensions were directly incubated with fluorochrome-labeled antibodies (Supplemental Table S1). For intracellular staining, cells were firstly fixed with the FIX&PERM™ Cell Permeabilization Kit Reagent A (Life Technologies) for 15 min at room temperature (RT) and then permeabilized with Reagent B and simultaneously incubated with the intracellular-labeled antibodies (20 min, RT). Cells were analyzed with FACSCalibur flow cytometer in CellQuest software.

### Indirect co-culture

EpSlKCs were harvested from the feeder layer [21], resuspended in KSFM with Y-27632, and seeded at a density of 500 cells/cm^2^. Co-cultures were established in the next day, using Transwell® inserts (0.4 μm pore, Corning). DP cells were plated in the insert at a density of 1000 cells/cm^2^. The co-culture medium consisted of an equal mixture of DMEM 10% FBS and KSFM with Y-27632 (DMEM:KSFM). Single cell-type cultures were prepared as controls, by culturing cells in their regular medium and in the co-culture medium, the latter to control possible medium effects on the cells.

### DNA and alkaline phosphatase quantification

At each experimental time point, cells were washed with PBS and lysed with 0.01% SDS (NZYteck). DNA was quantified using the Picogreen™ dsDNA kit (Thermo Fisher Scientific), and active ALP was quantified using the ALP Fluorescent Detection Kit (Sigma-Aldrich), both following the manufacturer’s instructions.

### Growth factor quantification

Growth factors present in the supernatants of the 48 h homotypic cultures or co-cultures were quantified using VEGF ELISA Development Kit (Peprotech), BMP2 ELISA Development Kit (Peprotech), and PDGF-A DuoSet (R&D Systems), according to the manufacturer’s instructions. DNA values were used to normalize results.

### Hair reconstitution assay

Athymic male nude Balb/C mice (Charles River, 9 weeks) were randomly divided into three experimental groups: control, DP cells, and DP cells with EpSlKCs (*n* = 4, each group). The silicone chamber model was set and used as previously described [22]. Cell suspensions with 5.0 × 10^6^ DP cells and 2.5 × 10^6^ EpSlKCs (experimental group), 5.0 × 10^6^ DP cells (DP cell group), or vehicle (FAD medium, control group) were injected in the chamber. The top of the chamber was cut-off after 1 week, and the chamber was fully removed after 2 weeks. Animals were euthanized 6 weeks after surgery to harvest the wound area for histological analysis.

### Immunofluorescence and histological analysis

Cells from the in vitro experiments were washed in PBS and fixed in 10% (v/v) formalin for 15 min (RT). Cell permeabilization was performed with 0.2% (v/v) Triton X-100 for 15 min (RT, intracellular staining) or 30 min (4 °C, nuclear staining), and blocking with a 3% (w/v) BSA solution for 30 min (RT). Samples were then incubated with primary antibodies (Supplemental Table S2) diluted in 1% BSA for 1 h (RT). After washing with PBS, Alexa Fluor (594/488)-conjugated secondary antibodies (1:500; Molecular Probes) were applied. Cellular morphology was observed after staining F-actin with Phalloidin-TRITC (1:100, Sigma-Aldrich) for 1 h (RT) and nuclear counterstain performed using DAPI (0.02 mg/ml, Biotium) for 15 min (RT). Images were acquired with an Axio Imager Z1m microscope (Zeiss).

For histological analysis, 4-μm paraffin-embedded sections were stained with hematoxylin and eosin (H&E), according to standard procedures, and analyzed with a DM750 microscope (Leica). For immunofluorescence studies, heat-mediated antigen retrieval was performed in the deparaffinized sections using the citrate buffer (pH 6.0), and the remaining procedure was performed as above described for the in vitro ones.

### Statistical analysis

Statistical analysis was performed using GraphPad Prism 7.03. To test if data followed a Gaussian distribution, the D’Agostino and Pearson normality test was used. Nonparametric data were analyzed with a Mann-Whitney *t* test (two groups, unpaired); the Kruskal-Wallis (three groups, unpaired) or Friedman test (three groups, paired) was used coupled with Dunn’s post-test. Parametric data were analyzed using a one-way ANOVA (two groups, paired) or RM two-way ANOVA (three groups, paired) in combination with Tukey’s post-test. Differences with *p* < 0.05 were considered significant.

## Results

### EpSlKCs and HHFKCs are phenotypically similar

**Fig. 1:**
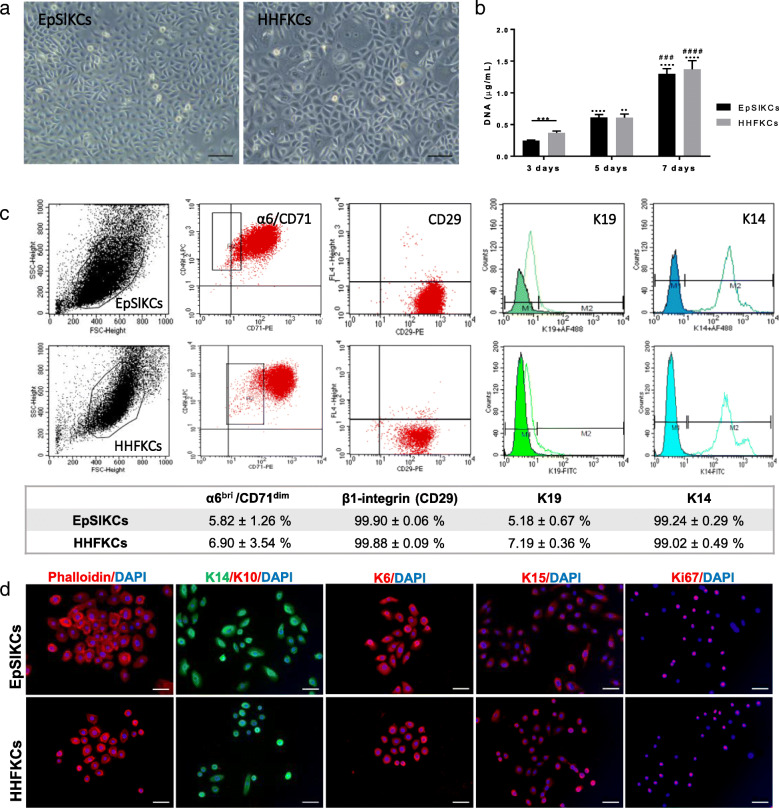
Morphology, proliferation, and phenotype of EpSlKCs and HHFKCs. **a** Representative light microscopy images of human epidermal stem-like keratinocyte (EpSlKC) and human hair follicular keratinocyte (HHFKC) culture. **b** DNA quantification of the cells along the culture time (*n* = 5, EpSlKCs; *n* = 4, HHFKCs). **c** Representative flow cytometry histograms and respective quantification regarding the percentage of α6^bri^/CD71^dim^ cells, and those positive for integrin β1 (CD29), keratin 19 (K19), and keratin 14 (K14) in EpSlKC (*n* = 4) and HHFKC (*n* = 3) cultures after 1 week. **d** Immunofluorescence staining of β-actin filaments (phalloidin), keratin 14 (K14), keratin 10 (K10), keratin 6 (K6), keratin 15 (K15), and the proliferation-associated marker Ki67 in EpSlKCs and HHFKCs. DAPI was used as a nuclear counterstainer. Data shown are mean ± SEM. Scale bars are 100 μm for **a** and 50 μm for **d**. ***p < 0.001; **··***p* < 0.01 and **····***p* < 0.0001 vs. day 3; ^###^*p* < 0.001 and ^####^*p* < 0.0001 vs. day 5

The isolated interfollicular KCs comprehended a low percentage of epidermal stem cells (4.62 ± 1.47%; α6^bri^/CD71^dim^ fraction) and differentiated cells (3.72 ± 0.47%; α6^dim^ subpopulation), while TA cells (78.44 ± 3.26%; α6^bri^/CD71^bri^ subpopulation) represented the majority of the population (Supplemental Fig. S1a), as expected [18]. The selected α6^bri^/CD71^dim^ cells were cultured on feeders (Supplemental Fig. S1b,c), and the obtained cells—EpSlKCs—were directly compared to HHFKCs. Most EpSlKCs and HHFKCs were small and bright cells displaying a cobblestone morphology (Fig. 1a), characteristic of undifferentiated epithelial cells. However, cellular heterogeneity was higher for HHFKC cultures, with the presence of large size cells, representative of differentiated cells. Nevertheless, both cell types proliferated at similar rates (Fig. 1b), although at day 3 HHFKC numbers were higher than EpSlKCs. The percentage of α6^bri^/CD71^dim^ cells in both cell types was similar, as was the expression of the basal epidermal markers integrin β1 (CD29) and keratin (K) 14 (Fig. 1c) . The expression of K19, typically considered a stem cell marker whose expression decreases with age [23], was also similar among cell types. Immunocytochemistry analysis confirmed their immature phenotype, with positive staining for the basal-specific markers K15, K6, and K14, and absence of the differentiation marker K10 (Fig. 1d). Additionally, most cells were positive for the proliferation-associated marker ki67. Together, these results demonstrate that EpSlKC and HHFKC proliferative capacity and phenotype are equivalent.

### EpSlKCs support DP cell growth and a partial restoration of their native phenotype

**Fig. 2:**
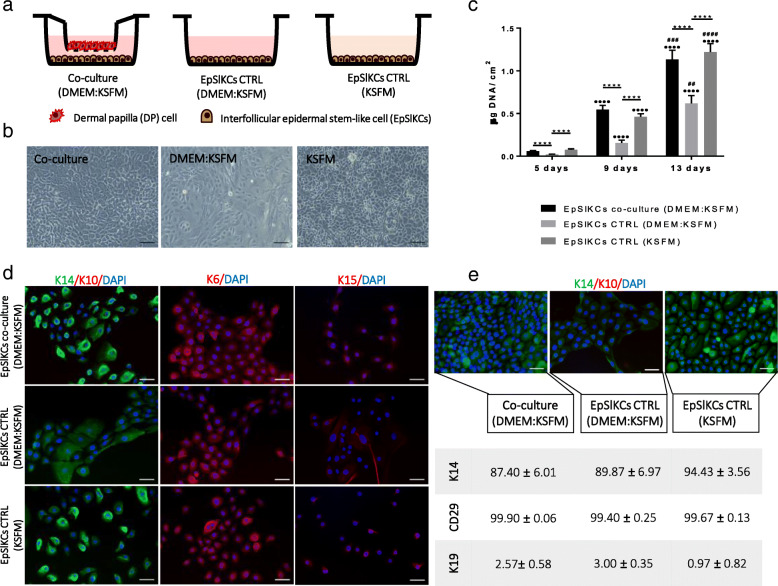
Characterization of EpSlKCs co-cultured with DP cells. **a** Schematic representation and **b** light microscopy images of EpSlKCs in co-culture with dermal papilla (DP) cells and in the respective monoculture controls. **c** DNA quantification of EpSlKCs after 5, 9, and 13 days of culture (*n* = 5). **d** Representative images of immunofluorescence staining against the epithelial markers K14 and K10, K6, and K15 after 5 days in culture. **e** Immunostaining against K14 staining in EpSlKCs after 13 days in culture and the respective flow cytometry quantitative data (*n* = 3). DAPI was used as nuclear counterstaining. Data shown are mean ± SEM. Scale bars are 100 μm for **b** and 50 μm for **d** and **e**. ****p < 0.0001; **····***p* < 0.0001 vs. day 5; ^##^*p* < 0.01, ^###^*p* < 0.001, and ^####^*p* < 0.0001 vs. day

To study EpSlKC capacity to communicate with DP cells, an indirect co-culture was established (Fig. 2a). After co-culture, EpSlKCs remained small and exhibited a cuboidal morphology (Fig. 2b). In the conventional culture medium (KSFM), EpSlKCs displayed a cobblestone morphology and were slightly brighter and smaller than in co-culture. Despite this, co-cultured EpSlKCs were different from those kept in the co-culture medium (DMEM:KSFM), whose morphology, larger size, and lower brightness evidenced a more differentiated state. Co-culture with DP cells also prevented the differentiation of EpSlKCs, expected to be induced by DMEM components, such as calcium [24, 25]. Furthermore, co-culture conditions were able to sustain EpSlKC proliferation along culture, as demonstrated by the similar DNA amounts of the co-culture and KSFM, both significantly higher than in the medium control (Fig. 2c). Further evaluation of DP cell effects on EpSlKC differentiation showed that K14 expression was similar in the co-cultures and in the monoculture established with KSFM. In contrast, K14-bright-positive cells were not found in the monocultures of EpSlKCs in the control medium (Fig. 2d). Nevertheless, K6 and K15 staining were similar among conditions, with cells clearly stained for K6 but weakly expressing K15. After 13 days in co-culture, most cells were K14-positive, as confirmed by flow cytometry analysis, but K14-bright cells were only observed for EpSlKCs cultured in KSFM (Fig. 2e). Independently of the culture conditions, EpSlKCs retained β1-integrin expression while K19 expression was almost absent.

**Fig. 3:**
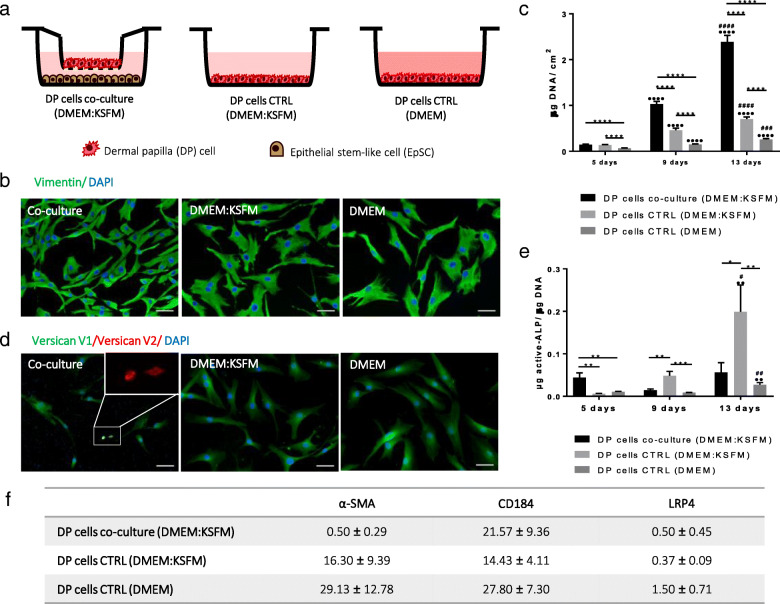
Characterization of DP cells co-cultured with EpSlKCs. **a** Schematic representation of the co-culture established with DP cells and EpSlKCs and the respective monoculture controls. **b** Representative images of DP cell morphology after staining against the mesenchymal cytoskeleton marker vimentin. **c** DNA quantification of DP cells along the culture time (*n* = 5). **d** Expression of the versican V1 and V2 isoforms by DP cells in the different culture conditions at day 5 of culture. The presence of versican V2-positive cells was observed when DP cells were co-cultured with EpSlKCs (zoomed-in image). Images were counterstained with DAPI. **e** The inductivity of DP cells was assessed through the quantification of active alkaline phosphatase (ALP, *n* = 3). **f** Quantitative flow cytometry data (*n* = 3). *p < 0.05, **p < 0.01, ***p < 0.001, ****p < 0.0001; **··***p* < 0.01 and **····***p* < 0.0001 vs. day 5; ^#^*p* < 0.05, ^##^*p* < 0.01, and ^####^*p* < 0.0001 vs. day 9. Scale bars are 50 μm

In addition to the effect of the co-culture over EpSlKCs, we looked to potential alterations on DP cells (Fig. 3a). In DMEM, most of the cells exhibited a flattened and enlarged morphology, typical of higher passage cells, while in co-culture they were smaller, with a polygonal or spindle-shaped morphology (Fig. 3b), evidencing a healthier state [26, 27]. When cultured in the control medium, DP cells had an intermediate morphology. Likewise, the cell number at each time point was successively higher from DMEM to the co-culture conditions revealing an increased proliferation capacity both due to the culture medium and the presence of EpSlKCs (Fig. 3c). The expression of the V1 isoform of versican was uniform among conditions, whereas the in vivo predominant V2 isoform [28] was only observed in co-cultured DP cells (Fig. 3d). Moreover, the amount of active alkaline phosphatase (ALP) in co-culture was significantly higher than that in the controls after 5 days in culture, although this effect was not sustained over time (Fig. 3e). A decrease in α-SMA expression was also observed in co-culture, although C-C-X-C chemokine receptor type 4 (CD184) and low-density lipoprotein receptor-related protein 4 (LRP-4) expression was not restored (Fig. 3f).

### EpSlKC and DP cell co-culture impacts the release of EMI mediators

To study if DP cells and EpSlKCs could communicate through the release of mediators, as seen in the native HF EMIs, we studied the release of PDGF-A, VEGF, and BMP2, known to influence hair growth [29–31].

**Fig. 4:**
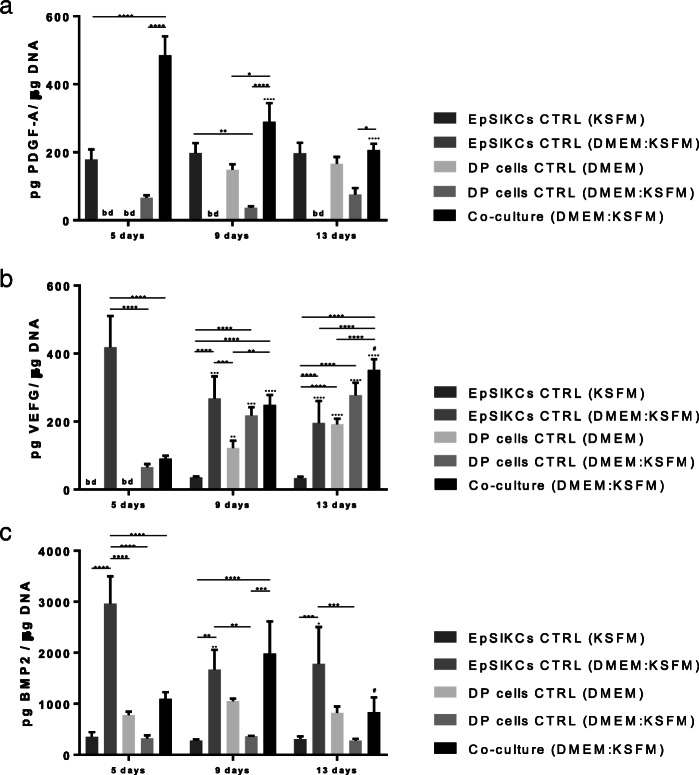
Quantification of the amount of **a** PDGF-A, **b** VEGF, and **c** BMP2 released by both co-cultured cells and monocultured EpSlKCs and DP cells by ELISA (*n* = 3). Data shown are mean ± SEM. *p < 0.05, **p < 0.01, ***p < 0.001, ****p < 0.0001; **∙** *p* < 0.05, **··** *p* < 0.01, **···** *p* < 0.001, **····** *p* < 0.0001 vs. day 5; ^#^*p* < 0.05 vs. day 9. bd, bellow detection level; BMP2, bone morphogenetic protein 2; PDGF-A, platelet-derived growth factor A; VEGF, vascular endothelial growth factor

PDGF-A production was higher in the co-culture than in the other conditions although successively lower levels were detected along the culture (Fig. 4a). EpSlKCs in KSFM were also able to secrete PDGF-A, which was at a constant level along the culture time. Secretion of PDGF-A by DP cells was only detected at 9 and 13 days of culture in DMEM. However, in the monocultures established to control medium effects (DMEM:KSFM), PDGF-A values were below the detection level for EpSlKCs and lower than the standard culture for DP cells from day 9 onward. Considering this, it seems that the interaction between EpSIKCs and DP cells in co-culture promotes PDGF-A production, particularly at early time points.

VEGF levels in the co-culture increased with time, and both EpSlKCs (*p* < 0.05) and DP cells secreted more VEGF when cultured in the medium used for the co-culture (DMEM:KSFM) than in KSFM and DMEM, respectively (Fig. 4b). Moreover, the amount of VEGF secreted by EpSlKCs decreased along the culture and increased for DP. Despite this, VEGF levels in the co-cultures and in the monocultures of DP cells in the same medium were similar independently of the time of culture. Thus, except at day 5 at which the VEGF amount was significantly lower in the co-culture than in the EpSlKC cultures established in the co-culture medium, it seems that the co-culture does not affect VEGF secretion.

In what concerns BMP2 secretion, the levels detected in the co-culture peaked on day 9 (Fig. 4c). The amount of BMP2 in the monocultures of EpSlKCs in KSFM was lower than that in the monoculture of DP cells in DMEM, and none varied with time. However, the medium used to establish the co-culture medium had contrary effects on each cell type, respectively promoting and diminishing BMP2 secretion by EpSlKCs and DP cells. Except for day 9, BMP2 levels in the co-cultures were always lower than those in the monocultures of EpSlKCs established with the same medium. This indicates that cells in co-culture are communicating, and suggests that DP cells may be responsible for the overall decreased BMP2 production in co-culture.

### Generation of HF and sebaceous gland-like structure by co-grafted EpSlKCs and DP cells

**Fig. 5:**
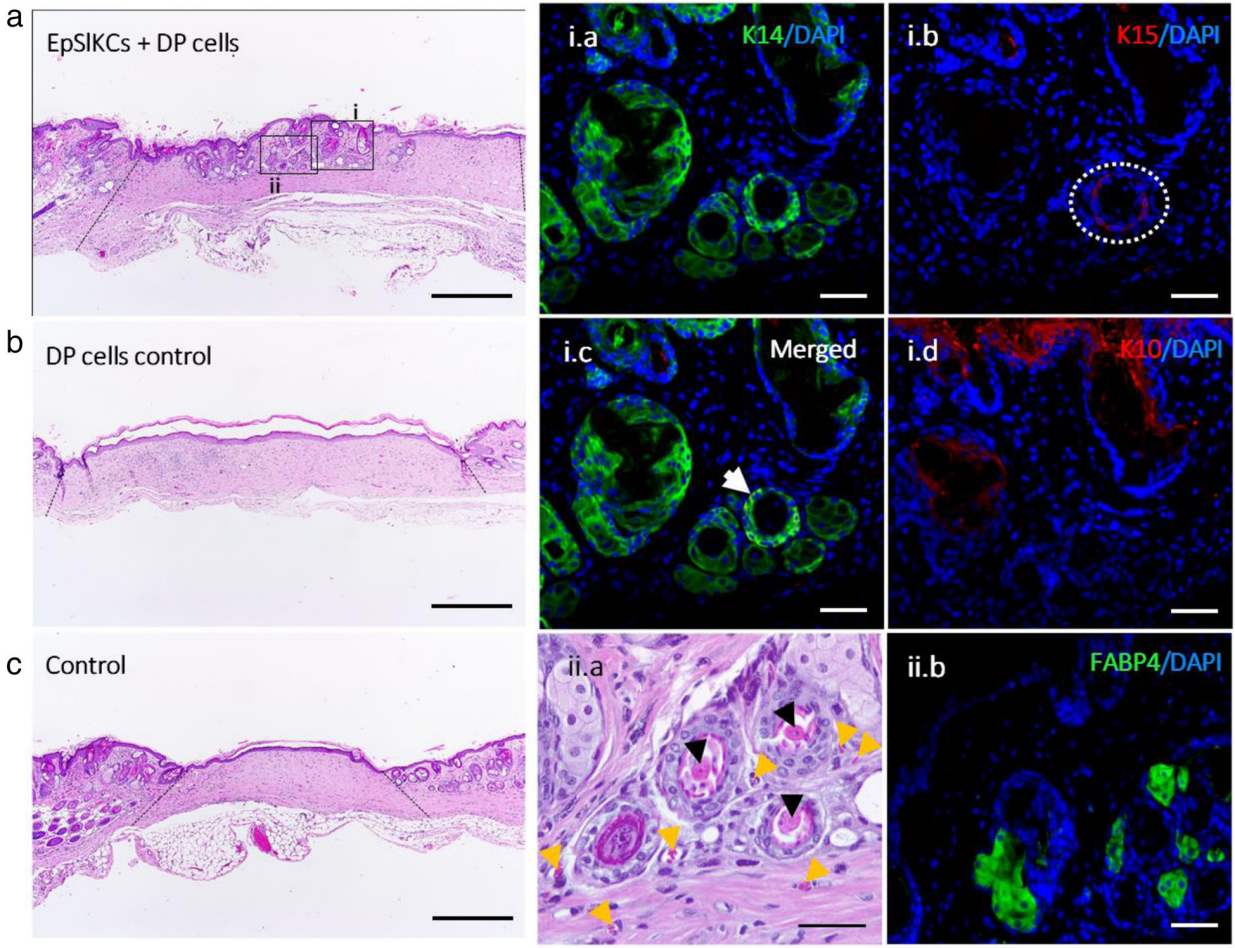
HF and SG induction in mice. **a** Representative hematoxylin and eosin (H&E) images of the area where EpSlKCs and DP cells, **b** DP cells alone, or **c** vehicle were transplanted (dashed lines indicating the wound area). Immunostaining of the recreated hair follicle (HF)- and sebaceous gland (SG)-like structures against the epithelial markers **i.a** K14, **i.b** K15 (dashed circle), or **i.c** both (white arrow). **i.d** Expression analysis of the differentiation marker K10 within the wound area (**ii.a**). H&E higher magnification image evidencing an apparently differentiated core (black arrowheads) within the formed structures, and the presence of small vessels around them (yellow arrowheads). **ii.b** Immunodetection of the fatty acid-binding protein 4 (FABP4) within the structures resembling SGs. Scale bars are 500 μm for **a**–**c** and 50 μm for **i.a**–**i.d** and **ii.a** and **ii.b**

Six weeks after EpSlKC and DP cell transplantation in mice, we observed the formation of structures that morphologically resembled HF and sebaceous glands (SG) in 3 out of 4 animals (Fig. 5a). These structures were not observed in the controls (Fig. 5b, c). The immunohistochemical analysis of the recreated structures showed the presence of epithelial layers positive for the basal markers K14 and K15 (Fig. 5i.a,b), in some cases co-expressed (Fig. 5i.c, white arrow), whereas K10 was observed in the skin outermost layer and in some of the structures’ core (Fig. 5i.d). The presence of multiple concentric epithelial layers, some of them with an apparently keratinized core (Fig. 5ii.a, black arrowhead), was also observed. This suggests that the differentiation process was initiated but remained incomplete, since features of mature HFs, including hair shaft formation, were not observed. Moreover, small vessels were observed around the recreated structures (Fig. 5ii.a, yellow arrowhead), while in the SG-like structures the expression of the adipocyte marker FABP4 (Fig. 5ii.b) demonstrates sebocyte lineage differentiation. Labelling with a human-specific DNA probe failed to detect the presence of human cells within the recreated structures (Supplemental Fig. S2). Nevertheless, the absence of structures in the controls suggests that the human EpSlKCs were critical for HF and SG induction process.

## Discussion

Despite the advances in rodent HF regeneration, thought the combination of receptive-epithelial and inductive-mesenchymal cells, human HF regeneration remains elusive. Major hurdles reside in the lack of tissue sources capable of providing immature epithelial cells, and of suitable culture conditions capable of minimizing cell differentiation in vitro. Here, we propose the use of EpSlKCs as an alternative to HHFKCs for the recreation of the HF epithelial compartment. Compared to HHFKCs, EpSlKCs represent a more homogeneous cell fraction and larger numbers of EpSlKCs can be obtained from surgically discarded skin, enabling hair regeneration studies/therapies.

Despite their undifferentiated profile and high clonogenic capacity [19], using EpSlKCs for the recreation of the HF epithelial compartment depends on their capacity to crosstalk with DP cells. We showed that DP cells promoted an increase in EpSlKC numbers, which is in agreement with previous studies confirming that DP cells enhance the growth of co-cultured ORS cells [32, 33]. For longer culture times, EpSlKCs in co-culture increased in size and the K14-bright cells were lost, suggesting differentiation. Despite this, their proliferation was not affected, which seems to indicate that DP cells are tightly balancing EpSlKC division and differentiation. This is important considering that DP controls the number of bulb KCs [34], whose continuous proliferation and differentiation are required for hair growth. Another indicator of this control was the release of VEGF and BMP2 by co-cultured cells. Epidermal VEGF is mainly expressed in the upper-to-middle epidermal layers, supporting a positive correlation between VEGF release and epithelial cell differentiation [35, 36]. This is also supported by the higher amount of VEGF detected in EpSlKCs cultured in the co-culture medium, in which EpSIKCs have a more differentiated phenotype. Interestingly, VEGF levels in the co-culture increased with time, which may indicate that EpSlKC differentiation is occurring. However, their proliferative capacity remained unaffected, once again indicating that EpSlKC differentiation and proliferation is balanced by DP cells. Also, BMP expression is subtle among proliferating epithelial cells and higher in differentiating cells [37]. Herein we showed that BMP2 secretion in the co-cultures peaked at day 9 while decreasing in the EpSlKC monocultures established in the same medium, once again suggesting DP cell effect.

EpSlKCs also improved DP cell proliferation, an effect already reported for rodent KCs [38]. To this might have contributed the marked increase in PDGF-A levels observed in the co-culture, particularly up to 9 days of culture. PDGF-A is a mitogenic factor typically produced by epidermal [39] and follicular keratinocytes [40], capable of stimulating mesenchymal cell proliferation [41]. Most importantly, the PDGF-A receptor is activated in the anagenic DP [42] and PDGF-A administration has been demonstrated to induce anagen [42, 43]. This suggests that PDGF-A increase in co-culture may not only promote DP cell proliferation but also stimulate their hair growth capacity. In fact, co-culture conditions’ positive effect on DP cell phenotype was demonstrated by the loss of α-SMA expression. Although commonly referred as a DP cell marker [44], α-SMA is only expressed in 2D-cultured DP cells and not in situ [45]; therefore, its near absence in the co-cultures seems an indicator that EpSlKCs are positively influencing DP cells. ALP activity and versican expression, in turn, are well-established markers of DP cell inductivity [44]. The presence of the V2 isoform of versican at early culture times, which is predominant in the native DP [28], and the increased number of ALP-active DP cells also suggest an improvement in the inductivity. Nevertheless, ALP activity is known to vary along the HF cycle, and differences in DP spatial-temporal ALP activity might correlate with distinct functions along the cycle [44, 46]. Furthermore, the release profile of BMP2 also suggests a hair inductive profile by co-cultured DP cells. BMP signaling is an anagen negative regulator [31, 47], which needs to be counteracted for HF induction to occur [31, 48, 49]. Therefore, the lower BMP2 expression observed at day 5, associated with increased PDGF-A levels, suggest that EpSlKC-DP cell communication in vitro may be mimicking early anagen features. This is interesting considering previous reports where ORS cell co-culture with DP cells, precisely for 4–5 days, restored ORS cell trichogenic ability, with DP cells influencing their competence [50, 51]. They also demonstrated that previously co-cultured ORS cells promoted HF formation, whereas monocultured ORS cells failed to do it. Remarkably, we observed the recreation of HF- and SG-like structures upon co-grafting of EpSlKCs and human DP cells, regardless of not having been co-cultured, which suggests that EpSlKCs can acquire follicle-specific competence in vivo upon interaction with DP cells.

The capacity of interfollicular epidermal cells to differentiate into follicular structures was already demonstrated in the chamber model, after their combination with murine DP cells [17]. However, to our knowledge, this is the first study to demonstrate the recreation of HF- and SG-like structures upon grafting adult human interfollicular KCs and DP cells. Additionally, the capacity of human epithelial cells to generate HF-like structures is higher for neonatal cells than for adult or passaged cells [17], supporting the importance of EpSlKC immature state. We demonstrated that KSFM-cultured EpSlKCs, as performed before grafting, are kept in a high proliferative state. Moreover, these cells are characterized by high production of PDGF-A, a factor involved in HF formation, and low secretion of the anagen inhibitor BMP2, which may have contributed to the observed response. The recreated structures displayed common features of the native HF, including the presence of cell layers co-expressing K15 and K14, as observed in the bulge and outermost upper layer of the ORS, or only expressing K14 as in the ORS suprabasal layers and respective progeny [52]. Similarly, K10 expression in the HF is limited to the differentiated follicular portions adjacent to the epidermis [52]. This pattern was simulated in the recreated structures, as shown by the K10 expression in the innermost epithelial layer of the HF-like structures closer to the epidermis and its absence in the ones located deeper in the dermis. We also observed the presence of blood vessels around the structures, likely a consequence of EpSlKC and DP cell production of VEGF, essential to induce and maintain the microvasculature around the HF and in the dermal compartment [30, 53]. Nevertheless, the follicle-like structures failed to produce a hair shaft, demonstrating that hair formation remained incomplete. Furthermore, we did not detect human cells within the recreated structures or in the grafting area, which has potentially limited the required signaling for the complete hair formation. The absence of the transplanted cells is not completely unexpected considering that, unlike other models, the chamber assay represents a wound healing environment [4], with high cellular turnover. Moreover, although the chamber prevents the contribution of host cells for the initial regenerative process, dissociated human cells are easily replaced by host KCs after wound closure [54]. Nonetheless, the lack of structures in the controls demonstrates that human EpSlKCs were critical for hair neogenesis.

## Conclusions

Taken together, our results show that EpSlKCs are phenotypically similar to HHFKCs and can crosstalk and beneficially impact DP cells in vitro. When combined with cultured DP cells in vivo, EpSlKCs demonstrated their competence by promoting the recreation of structures that resembled the HF and SG initial morphogenetic events. Thus, EpSlKCs represent an alternative immature and available epithelial cell fraction for the recreation of the HF epithelial compartment.

### Supplementary Information

**Additional file 1: Fig. S1.** Phenotype of the CD49f^bri^/CD71^dim^ subpopulation. A) Representative image of fluorescent activated cell sorting (FACS) dotplot of the whole population of epidermal keratinocytes and the gatting of the subpopulations of cells, based on the expression of the CD49f and CD71 markers, accompanied by the respective cellular percentages. CD49f^bri^/CD71^dim^ cells represent epidermal stem-like cells, CD49f^bri^/CD71^bri^ subpopulation are transit-amplifying (TA) cells, while differentiated (Diff.) cells are characterized by a CD49f^dim^ phenotype. Epidermal stem-like cells derived colonies growing in the inactivated feeders (B) 4 and (C) 6 days after seeding. Data shown are mean ± SEM. Scale bars are 50 μm. **Fig. S2**. Human cell detection within skin explants from the hair reconstitution assay. Staining with a human-specific probe demonstrates that, 6 weeks after cells injection, there were no human cells remaining in the wound area in the controls (A,B) and in the condition where interfollicular epidermal stem-like keratinocytes (EpSlKCs) and DP cells were co-grafted **(C)**. Likewise, no staining was observed in the mice tissue (D), whereas nuclear orange-pink staining was observed in human positive control specimens (E,F), demonstrating the specificity of the staining. Scale bars are 200 μm, for (A-D) and 50 μm for (E,F). **Table S1.** List of antibodies used for flow cytometry studies. **Table S2.** List of antibodies used for immunofluorescence studies.

## Data Availability

The authors confirm that the data supporting the findings of this study are available within the article and its supplementary materials.

## Abbreviations

ALP: Alkaline phosphatase
BMP2: Bone morphogenetic protein 2
CD184: C-X-C chemokine receptor type 4
CD29: Integrin β1
CD49f: Integrin α6
CD71: Transferrin receptor
DP: Dermal papilla
DMEM: Dulbecco’s modified eagle medium
EMIs: Epithelial-mesenchymal interactions
EpSlKCs: Epidermal keratinocytes with stem-like features
FABP4: Fatty acid-binding protein 4
FACS: Fluorescence-activated cell sorting
HF: Hair follicle
HHFKCs: Human hair follicular keratinocytes
K: Keratin
KSFM: Keratinocyte serum-free medium
KCs: Keratinocytes
LRP-4: Low-density lipoprotein receptor-related protein 4
ORS: Outer root sheath
PDGF-A: Platelet-derived growth factor A
SG: Sebaceous gland
SMA: Smooth muscle actin
TA: Transit-amplifying
VEGF: Vascular endothelial growth factor
