# Correction: Interfollicular epidermal stem-like cells for the recreation of the hair follicle epithelial compartment

**DOI:** 10.1186/s13287-023-03509-y

**Published:** 2023-10-21

**Authors:** Carla M. Abreu, Rogério P. Pirraco, Rui L. Reis, Mariana T. Cerqueira, Alexandra P. Marques

**Affiliations:** 1 3B’s Research Group – Biomaterials, Biodegradables and Biomimetics, Headquarters of the European Institute of Excellence on Tissue Engineering and Regenerative Medicine, University of Minho, Guimarães, Portugal; 2 ICVS/3B’s – PT Government Associate Laboratory, Braga/Guimarães, Portugal

**Correction: Stem Cell Research & Therapy (2021) 12:62** 10.1186/s13287-020-02104-9

The original article [1] mistakenly omitted Funding information. The correct Funding statement is as follows:

**Funding**

This study was supported by FCT – Fundação para a Ciência e a Tecnologia, I.P., component OE under the scope of project UIDB/50026/2020 and by FCT/MCTES (Fundação para a Ciência e a Tecnologia/Ministério da Ciência, Tecnologia, e Ensino Superior) through the PD/59/2013, PD/BD/113800/2015 (C.M. Abreu), CEECIND/00695/2017 (M.T. Cerqueira), IF/00347/2015 (R. P. Pirraco), and IF/00945/2014 (A.P. Marques) grants.
