# Supplementary material for: Interfollicular epidermal stem-like cells for the recreation of the hair follicle epithelial compartment
Source: Stem Cell Res Ther. 2021 Jan 15;12:62. doi: 10.1186/s13287-020-02104-9 (PMC7811263; doi:10.1186/s13287-020-02104-9)
Supplement: Supplementary file 1 — Additional file 1: Fig. S1. Phenotype of the CD49fbri/CD71dim subpopulation. A) Representative image of fluorescent activated cell sorting (FACS) dotplot of the whole population of epidermal keratinocytes and the gatting of the subpopulations of cells, based on the expression of the CD49f and CD71 markers, accompanied by the respective cellular percentages. CD49fbri/CD71dim cells represent epidermal stem-like cells, CD49fbri/CD71bri subpopulation are transit-amplifying (TA) cells, while differentiated (Diff.) cells are characterized by a CD49fdim phenotype. Epidermal stem-like cells derived colonies growing in the inactivated feeders (B) 4 and (C) 6 days after seeding. Data shown are mean ± SEM. Scale bars are 50 μm. Fig. S2. Human cell detection within skin explants from the hair reconstitution assay. Staining with a human-specific probe demonstrates that, 6 weeks after cells injection, there were no human cells remaining in the wound area in the controls (A,B) and in the condition where interfollicular epidermal stem-like keratinocytes (EpSlKCs) and DP cells were co-grafted (C). Likewise, no staining was observed in the mice tissue (D), whereas nuclear orange-pink staining was observed in human positive control specimens (E,F), demonstrating the specificity of the staining. Scale bars are 200 μm, for (A-D) and 50 μm for (E,F). Table S1. List of antibodies used for flow cytometry studies. Table S2. List of antibodies used for immunofluorescence studies. [file 13287_2020_2104_MOESM1_ESM.docx]

**Supplemental Materials and Methods**

**Chromogenic in situ hybridization**

The presence of human cells within the wound area was assessed using a human-specific DNA oligo probe and the respective detection system (BIO-HRP REMBRANDT® Universal DISH detection kit, PanPath). Briefly, dewaxed sections were blocked with a 3% solution of hydrogen peroxide (15min, RT) and proteolytic digestion was performed using a pepsin-HCL solution for 30min at 37ºC, followed by the dehydration in graded ethanol series. The specimens were then airdried and 1 drop of the probe was applied and covered with a coverslip. Denaturation was performed at 95ºC for 5min, and hybridization for 16 hours at 37ºC in a moisturized environment. Samples were then washed in TBS buffer and incubated for 10 min with the stringency wash buffer (PanWash). After rinsing with TBS, the detection was performed by incubation with the BIO-HRP conjugate (30min, 37ºC). The samples were then washed 3 times in TBS and water and the chromogenic detection was performed using the kit AEC substrate detection system, with color development for 10 min at 37ºC (dark). The reaction was stopped with water and the samples observed under a DM750 light microscope (Leica).

**S****upplemental Tables**

**Table S1 -** List of antibodies used for flow cytometry studies

| **Cell type** | **Antibody** | **Reference** | **Brand** | **Dilution** | **Type of staining** |
| --- | --- | --- | --- | --- | --- |
| **HHFKs and**  **EpSlKCs** | CD71-PE | 334106 | BioLegend, USA | 1:66 | Direct, Surface staining (30min, RT) |
|  | CD49f-APC | 17-0495-82 | eBiosciences, USA | 1:33 |  |
|  | CD29-PE | 555443 | BD Biosciences, USA | 1:33 |  |
|  | K14-FITC | MCA890F | Bio-Rad, USA | 1:50 | Direct, Intracellular Staining |
|  | K19-AF488 | A4-120-C100 | ExBio, Czech Republic | 1:20 |  |
| **DP cells** | CD184 -APC | 306510 | BioLegend, USA | 1:20 | Direct, Surface staining (30min, RT) |
|  | LRP4-PE | 306510 | [Miltenyi Biotec](https://www.miltenyibiotec.com/ds/130-109-183), Germany | 1:50 | Direct, Surface staining (10min, ice) |
|  | αSMA-FITC | ab8211 | Abcam, UK | 1:33 | Direct, Intracellular Staining |

**Table S2 -** List of antibodies used for immunofluorescence studies

| **Antibody** | **Supplier** | **Host specie** | **Type** | **Dilution** |  | **Reference** |
| --- | --- | --- | --- | --- | --- | --- |
| Cytokeratin 14 | BioLegend (USA) | Rabbit | Polyclonal | 1:800 |  | PRB-155P |
| Cytokeratin 10 | Abcam (UK) | Mouse | Monoclonal | 1:100 |  | Ab9026 |
| Cytokeratin 6 | Abcam (UK) | Mouse | Monoclonal | 1:200 |  | Ab75703 |
| Cytokeratin 15 | Abcam (UK) | Mouse | Monoclonal | 1:50 |  | Ab1385 |
| ki67 | Abcam (UK) | Rabbit | Monoclonal | 1:50 |  | Ab16667 |
| Vimentin | Abcam (UK) | Rabbit | Monoclonal | 1:50 |  | Ab92547 |
| Versican V1 | Abcam (UK) | Rabbit | Polyclonal | 1:200 |  | Ab19345 |
| Versican V2 | DSHB (USA) | Mouse | Monoclonal | 1:10 |  | 12C5 |
| FABP4 | Abcam (UK) | Rabbit | Polyclonal | 1:100 |  | Ab66682 |

**Supplemental Figures**

**
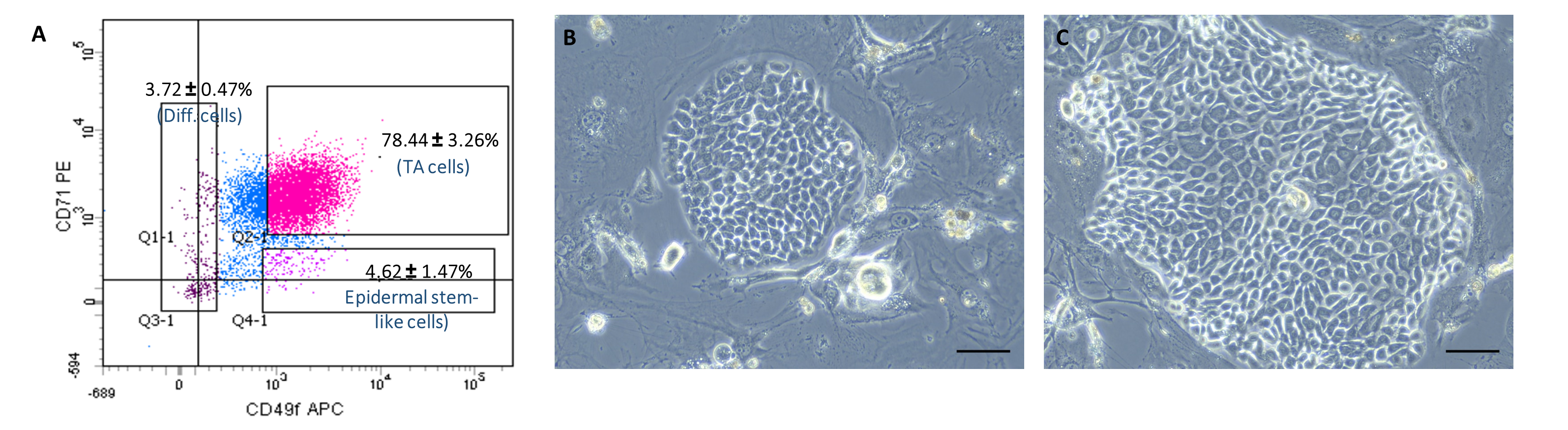
**

**Fig. S1- Phenotype of the CD49f^high^/CD71^dim^ subpopulation. (A)** Representative image of fluorescent activated cell sorting (FACS) dotplot of the whole population of epidermal keratinocytes and the gatting of the sub-populations of cells, based on the expression of the CD49f and CD71 markers, accompanied by the respective cellular percentages. CD49f^high^/CD71^dim^ cells represent epidermal stem-like cells, CD49f^high^/CD71^high^ subpopulation are transit-amplifying (TA) cells, while differentiated (Diff.) cells are characterized by a CD49f^dim^ phenotype. Epidermal stem-like cells derived colonies growing in the inactivated feeders **(B)** 4 and **(C)** 6 days after seeding. Data shown are mean ± SEM. Scale bars are 50μm.


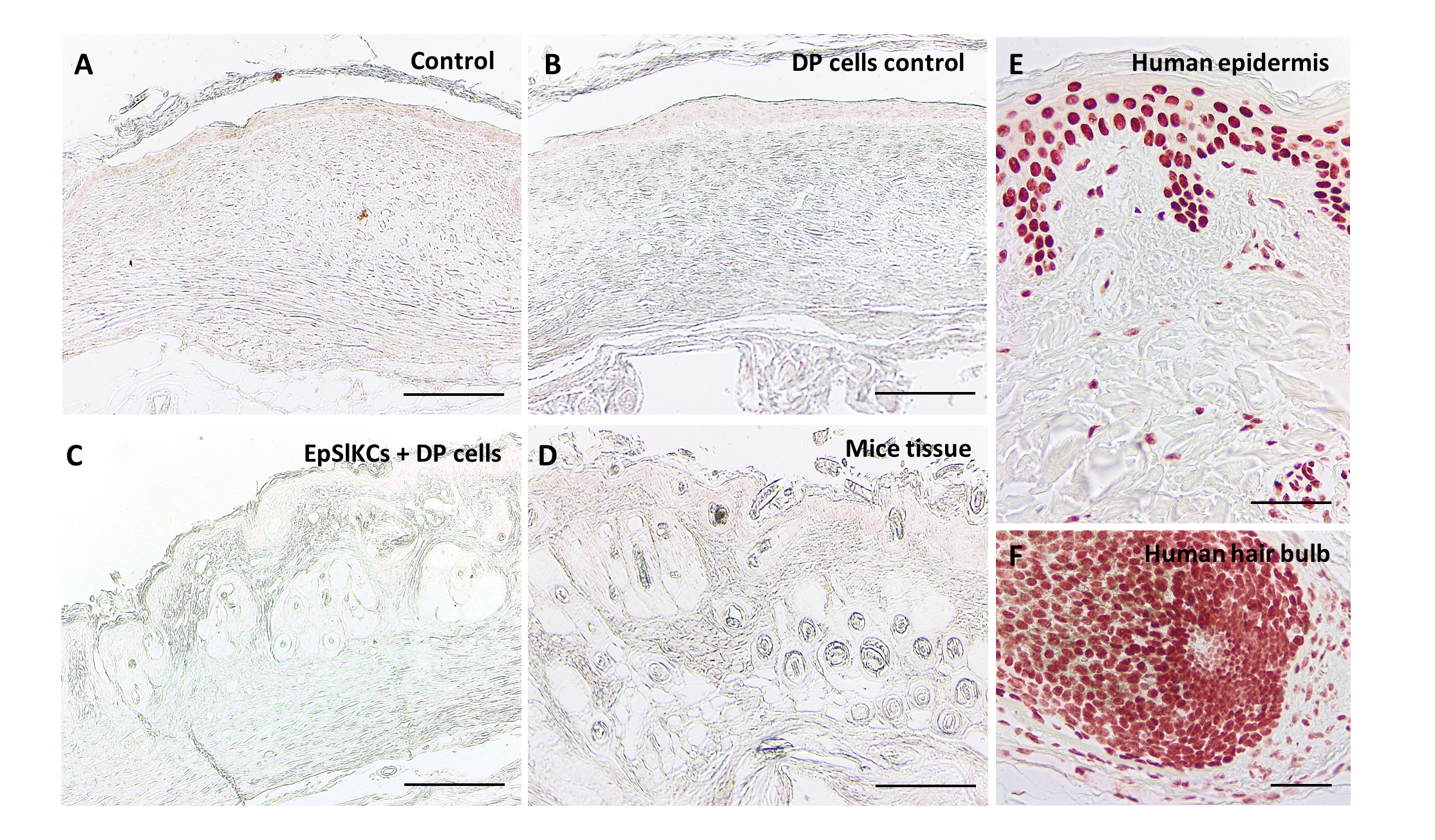


**Fig. S2- Human cell detection within skin explants from the hair reconstitution assay.** Staining with a human-specific probe demonstrates that, 6 weeks after cells injection, there were no human cells remaining in the wound area in the controls **(A,B)** and in the condition where interfollicular epidermal stem-like keratinocytes (EpSlKCs) and DP cells were co-grafted **(C)**. Likewise, no staining was observed in the mice tissue **(D)**, whereas nuclear orange-pink staining was observed in human positive control specimens **(E,F)**, demonstrating the specificity of the staining. Scale bars are 200μm, for **(A-D)** and 50μm for **(E,F)**.
